# Characteristics of Abdominal Movement Signals Measured by a Wireless Abdomen-Worn Sensor During Home Sleep Apnea Testing: Pilot Observational Study

**DOI:** 10.2196/93351

**Published:** 2026-09-01

**Authors:** Thi Hang Dang, Nam-Hwan Sung, Hyung-ki Min, Seongmun Kim, Heein Yoon, Franklin Bien

**Affiliations:** 1 Department of Electrical Engineering, Ulsan National Institute of Science and Technology, 50, UNIST-gil, Ulsan, 44919, Republic of Korea, 82 10-5545-4800; 2 SB Solutions Inc., Ulsan National Institute of Science and Technology, Ulsan, Republic of Korea

**Keywords:** sleep apnea, thoracoabdominal movement, wearable device, abdomen-worn sensor, wireless sensor, home sleep apnea testing

## Abstract

**Background:**

Respiratory inductance plethysmography (RIP) belts are the standard for measuring thoracoabdominal movements in home sleep apnea testing (HSAT), but are often cumbersome and power-intensive.

**Objective:**

This study aimed to characterize abdominal movement signals measured by a wireless, single-point, abdomen-worn sensor to evaluate the sensor’s capability to track respiratory dynamics.

**Methods:**

Overnight recordings were obtained from 37 participants using a wireless abdomen-worn sensor and a thoracic RIP belt during HSAT. The abdominal movement signal was analyzed for breath detection, respiratory rate (RR) estimation, and waveform similarity relative to the RIP signal. Factors influencing the agreement between the 2 signals were also investigated.

**Results:**

Data from 34 participants were analyzed. The abdominal movement signal showed moderate agreement with the thoracic RIP signal, achieving a sensitivity of 82.44%, a positive predictive value of 76.22%, and an *F*_1_-score of 78.99% for breath-cycle detection. RR estimation yielded a mean absolute percentage error of 5.42% and limits of agreement of ±3 breaths per minute (bpm). Morphological similarity was moderate, with an average distance correlation of 0.73 and a mean squared error of 0.64. The agreement between the 2 signals declined with increasing respiratory event severity.

**Conclusions:**

Compared with a standard thoracic RIP belt, the single-point, wireless, abdomen-worn sensor tracked basic respiratory metrics and waveform morphology with moderate agreement. These findings show promising baseline performance and suggest its viability as a simplified, low-profile data-acquisition platform for home-based respiratory monitoring.

## Introduction

Sleep apnea (SA) is characterized by repetitive episodes of airflow reduction (hypopnea) or cessation (apnea) for at least 10 seconds during sleep and is associated with intermittent hypoxia [1]. SA is highly associated with cardiovascular disease, affecting more than 40% of patients with these conditions [2]. Although SA increases the risk of heart failure by 140%, stroke by 60%, and coronary heart disease by 30%, SA is often undiagnosed and untreated in cardiovascular practice [2,3]. Thus, accurate diagnosis and appropriate management of SA are critical for mitigating cardiovascular risk. The gold standard method for diagnosing SA is polysomnography (PSG), which is expensive and has limited accessibility. Recently, home sleep apnea testing (HSAT) has gained widespread adoption for detecting SA because of its convenience and cost-effectiveness compared with PSG [4,5]. A typical HSAT configuration includes nasal pressure sensors, respiratory inductance plethysmography (RIP) belts, and pulse oximetry [5]. RIP belts encircle the torso and measure respiratory movements by detecting changes in band expansion during breathing [5]. Thoracoabdominal RIP belts are the recommended sensors by the American Academy of Sleep Medicine for apnea event classification in nocturnal PSG [6,7]. However, thoracoabdominal measurements using RIP belts can be cumbersome and are often associated with high power consumption [8,9].

To overcome the limitations of RIP belts, various alternative approaches have been proposed, including both contactless and contact-based measurement techniques, such as cameras, radar, and strain sensors [10-12]. However, the accuracy of contactless methods is significantly influenced by environmental factors, making them less suitable for HSAT. A promising approach to overcome the drawbacks of both contactless and conventional contact-based methods is the use of a single-point mechanical contact sensor placed on the thorax, abdomen, or neck for thoracoabdominal measurements. Among the single-point sensors, tracheal microphones and accelerometer-based sensors have been widely investigated due to their simplicity and low power consumption [13-17]. Although studies have shown that the thoracic RIP signal can be estimated from acceleration and suprasternal pressure (captured by tracheal microphones), these signals are proxy measures of thoracoabdominal movement. As a result, accurately approximating the thoracic RIP from these signals often requires complex processing techniques, such as principal component analysis or deep learning–based methods. Additionally, neck-mounted sensors are generally more prone to artifacts from head, neck, or body movements during sleep compared to sensors placed on the chest or abdomen.

In a previous study, we introduced an abdomen-worn sensor system (Soomirang) as a promising tool for SA screening [18]. This sensor operates passively without requiring an external power source, providing a simple and energy-efficient solution for monitoring thoracoabdominal movement [19,20]. It detects thoracoabdominal movement by measuring capacitance changes resulting from variations in the distance between the sensor and the body surface during breathing. The sensor is susceptible to motion artifacts, particularly those arising from body shifts during sleep. To mitigate this limitation, a triaxial accelerometer was integrated into the system to detect and account for gross movements. Although our previous studies demonstrated the feasibility of SA screening using the wireless abdomen-worn sensor, either as a standalone device [18] or as part of an integrated multisensor system [19], the fundamental characteristics and baseline measurement fidelity of the abdominal movement (Abdo) signal itself have not been comprehensively reported, resulting in limited insight into the underlying detection mechanisms.

This study characterizes the Abdo signals acquired from a wireless abdomen-worn sensor over an entire night of HSAT, encompassing both normal and disordered breathing periods. The Abdo signals are compared with synchronized respiratory signals obtained from a thoracic RIP belt, which serves as the reference standard. The primary objective is to evaluate breath detection, respiratory rate (RR) estimation, and temporal waveform agreement between the Abdo and RIP reference signals across consecutive respiratory cycles. In addition, we examine the effects of respiratory events and demographic factors on the performance of the wireless abdomen-worn sensor.

## Methods

### Soomirang System

**Figure 1.**
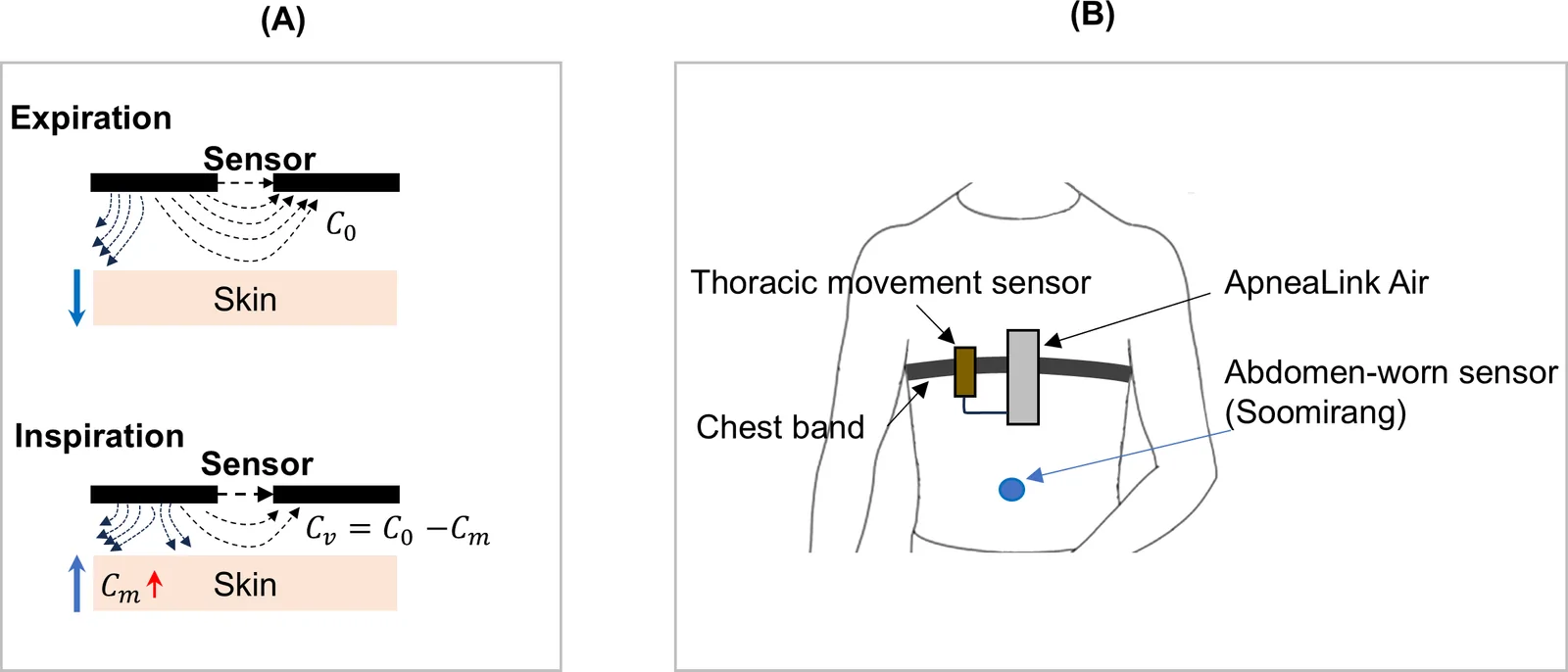
(A) Detection of surface displacement during inspiration and expiration based on the fringing electric field principle. (B) Simultaneous acquisition of thoracic movement using a thoracic respiratory inductance plethysmography (RIP) belt (reference signal) and abdominal movement using an abdomen-worn sensor.

The abdomen-worn sensor is a compact, 45-mm-diameter device designed for Abdo monitoring [19]. It integrates several key components, including a power module, Bluetooth module, capacitance-to-digital conversion system with a capacitive sensor, 3-axis accelerometer, and memory. The Bluetooth module enables real-time data communication with a smartphone app. The capacitive sensor embedded in the printed circuit board records respiratory movement signals. The capacitance-to-digital conversion system processes these analog signals, converts them into digital data, and transmits the resulting data via Bluetooth. Additionally, the 3-axis accelerometer detects directional movement and sends motion data to the Bluetooth module. In cases where Bluetooth connectivity is interrupted, data are temporarily stored in memory and transmitted once the connection is restored. The abdomen-worn sensor is placed on the abdomen or chest to detect surface movement based on the fringing field principle, where the electric field extending beyond the capacitor’s plates is influenced by the presence of the skin [19]. The capacitance variation (*C*_v_) is determined by the difference between the initial capacitance (*C*_0_) and the mutual capacitance (*C*_m_), which changes with the proximity of the skin. During inspiration, the expansion of the body surface decreases the distance between the skin and the capacitive sensor, increasing *C*_m_ and leading to a decrease in *C*_v_. Conversely, during expiration, contraction of the chest or abdomen increases the distance between the skin and the capacitive sensor, decreasing *C*_m_ and resulting in an increase in *C*_v_. Figure 1A illustrates how the abdomen-worn sensor detects surface movement during inspiration and expiration using the fringing electric field principle.

### Data Collection

Thirty-seven participants were recruited for the experiment. Written informed consent was obtained from all participants. All methods were performed in accordance with the ethical principles of the Declaration of Helsinki and were approved by the institutional review board of the Ulsan National Institute of Science and Technology (UNIST; UNISTIRB-24-006-A). Participant recruitment was carried out between March 2024 and April 2024. Table 1 summarizes the demographics of the participants in the study. Factors included sex, age, BMI, apnea-hypopnea index (AHI), and oxygen desaturation index (ODI). Each participant underwent overnight monitoring with simultaneous data collection using the ApneaLink Air device (AL, ResMed) and the Soomirang system. To provide a comfortable sleeping environment while complying with institutional safety requirements for an investigational device, the study was conducted in a dedicated residential-style testing suite at UNIST rather than in participants’ homes or a conventional hospital sleep laboratory. The suite was designed to simulate a home sleeping environment and was equipped with private sleeping partitions and portable beds. Throughout the overnight recording, research personnel remained in an adjacent room to monitor participant safety without interfering with the sleep study. The AL device was chosen for its proven reliability in detecting SA and its widespread use in HSAT [21,22]. The AL recorded oronasal airflow at a sampling frequency of 100 Hz, thoracic RIP at 10 Hz, and pulse and peripheral oxygen saturation at 1 Hz. The abdomen-worn sensor was positioned on the abdomen between the navel and the chest and captured the Abdo signal and 3-axis acceleration at 5 Hz. Figure 1B illustrates the setup of the thoracic RIP belt and the abdomen-worn sensor for the experiment.

**Table 1.** Demographics of the participants (N=37).

Variables	Values
Sex, n	
Females	10
Males	27
Age (y), mean (SD), range	37.33 (8.99), 27–65
BMI (kg/m^2^), mean (SD), range	26.85 (4.73), 18.0‐35.7
Apnea-hypopnea index (AHI; events/hour), mean (SD), range	23.42 (28.87), 0.4‐84.3
Oxygen desaturation index (ODI; events/hour), mean (SD), range	19.04 (22.46), 0‐72.1

### Data Processing

**Figure 2.**
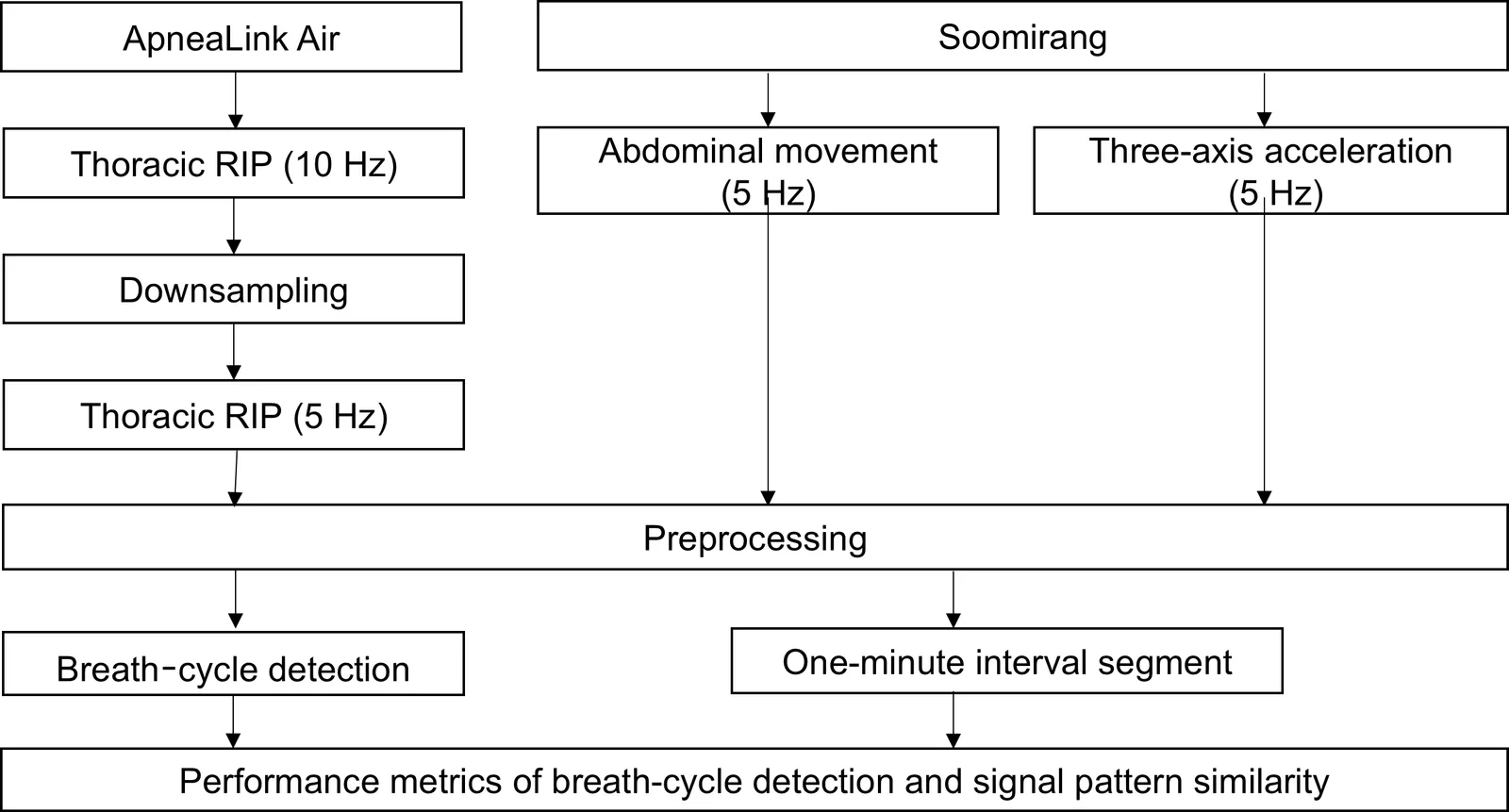
An overview of the methodology used to evaluate the abdominal movement signal in comparison to the thoracic movement signal. RIP: respiratory inductance plethysmography.

Figure 2 provides an overview of the methodology used to evaluate the Abdo signal compared with the thoracic movement signal recorded by the RIP belt. The evaluation focuses on 2 main aspects: breath-cycle detection performance and signal pattern similarity.

#### Preprocessing

The data obtained from the AL device and Soomirang system were time-synchronized. To ensure temporal alignment, only the overlapping recording interval between the Soomirang and AL was retained. The synchronized window (*T*) was defined as follows: *T*_start_=max(*T*_Soomirang, start_, *T*_AL, start_) and *T*_end_=min(*T*_Soomirang, end_, *T*_AL, end_), where *T*_Soomirang_ and *T*_AL_ represent the absolute timestamp arrays of the Soomirang and AL, respectively. Both data streams were then truncated to the common interval [*T*_start_, *T*_end_] by identifying the nearest boundary samples using the minimum absolute time-difference criterion. Breathing-disordered events were identified using AL software (version 10.10), which classified each sample as obstructive apnea (OA), central apnea (CA), mixed apnea (MA), hypopnea (H), or nonapnea (N). Each Soomirang data sample was then assigned a corresponding label of OA, CA, MA, or H if its timestamp fell within the duration of a matching event. Otherwise, it was labeled as N. To ensure consistency in the sampling rates, the thoracic RIP signal was downsampled from 10 to 5 Hz. Low-frequency baseline drift was removed from the Abdo signal using a causal, 1-sided moving-average detrending filter. The local baseline was continuously tracked using a causal sliding window comprising the current sample and the 55 preceding samples (11 s). The baseline-removed signal, $\overset{´}{x}[n]$, was obtained by systematically subtracting the estimated local baseline from the original signal, *x* [*n*], expressed mathematically as follows:


$$\overset{´}{x}\left[n\right]\text{=}x\left[n\right]-\frac{1}{M}\sum_{i\text{=}0}^{M-1}x\left[n-i\right]$$

**Figure 3.**
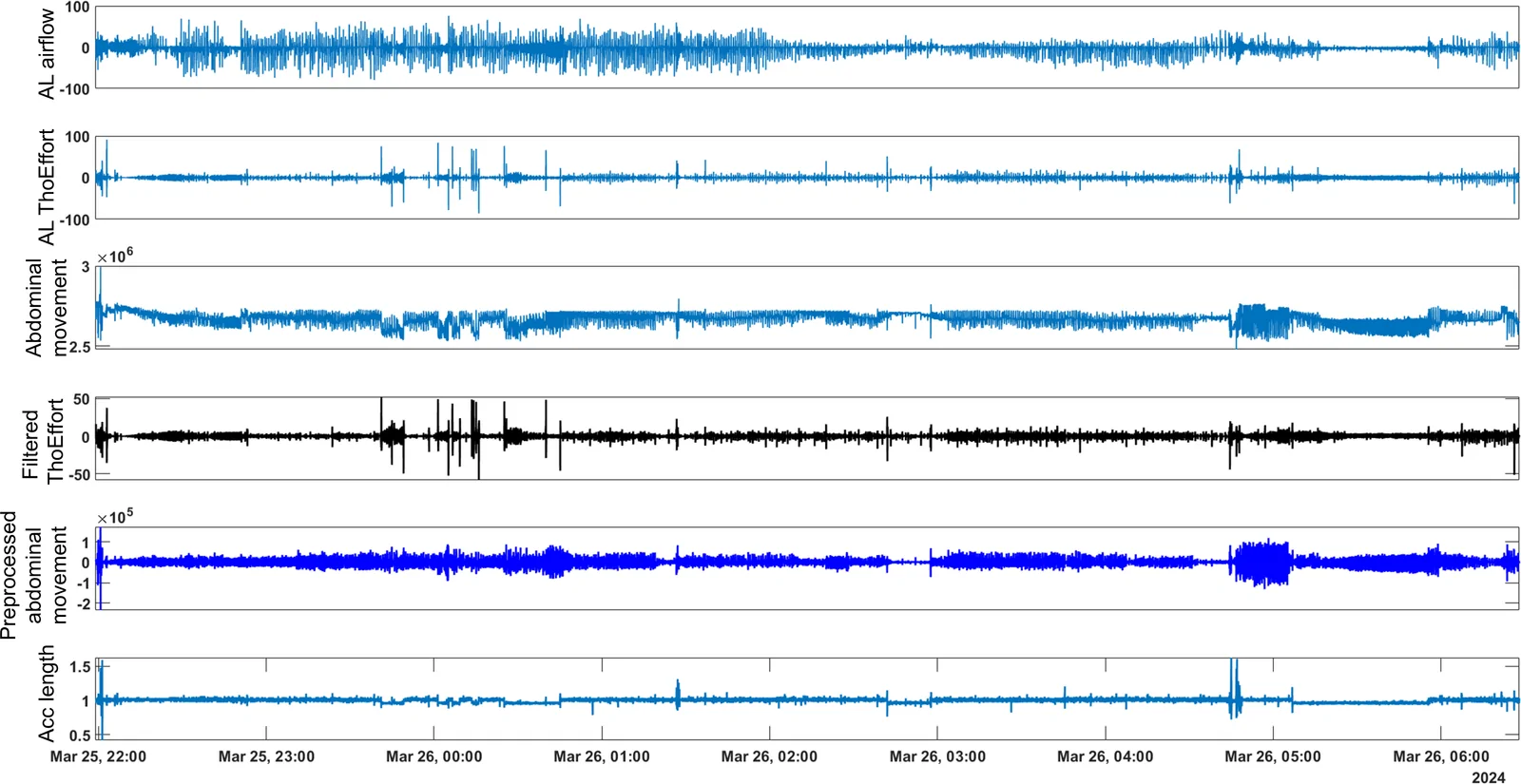
Measured signals from a full-night measurement using the ApneaLink Air (AL) device (AL airflow: airflow and AL ThoEffort: thoracic RIP) and the Soomirang system (Acc length: length of 3-axis acceleration and the abdominal movement signal). RIP: respiratory inductance plethysmography.

**Figure 4.**
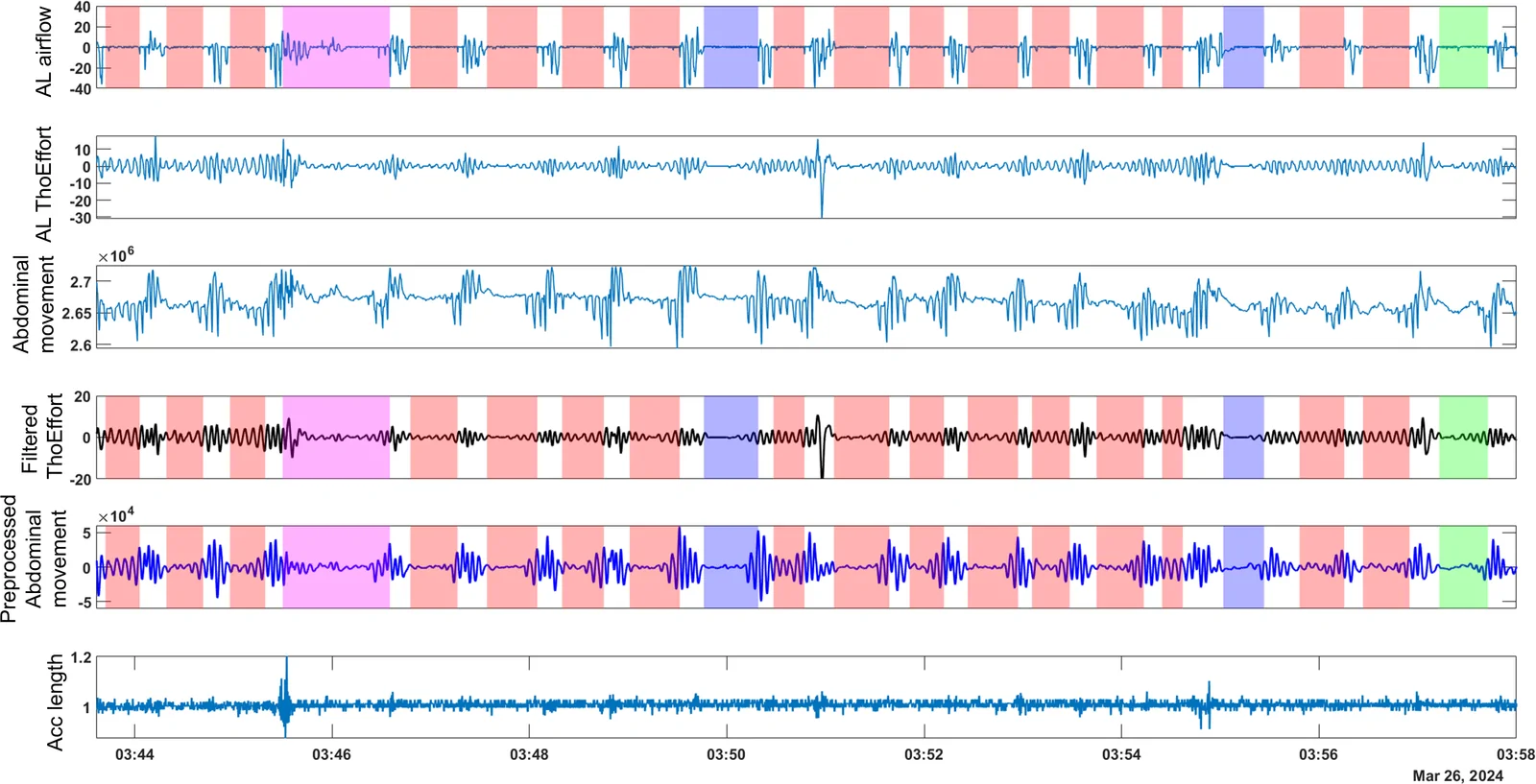
A detailed visualization of signals measured by ApneaLink Air (AL) device (AL airflow: airflow RIP and AL ThoEffort: thoracic RIP) and Soomirang system (Acc length: length of 3-axis acceleration and the abdominal movement signal), in which breathing-disordered events scored by the AL software are marked as follows: obstructive apnea (OA) events in red, hypopnea (H) in magenta, mixed apnea (MA) in green, and central apnea (CA) in blue. RIP: respiratory inductance plethysmography.

Here, *M* denotes the total causal window length. Both the Abdo and thoracic RIP signals were then filtered using a Savitzky-Golay filter with a window length of 11 samples and a filter order of 3 [23]. The length of the acceleration vector (Acc length) was calculated as the square root of the sum of the squares of the data from each axis. The rolling SD of the Acc length (σ_acc_) was calculated by calculating the SD during a moving window of 5 samples to capture local variations per second. Figure 3 shows the measured signals during a full-night measurement from the AL device and Soomirang system. Figure 4 shows a detailed visualization of signals with breathing-disordered events scored by AL software.

#### Breath-Cycle Detection

A customized peak detection algorithm was used to identify the peaks and valleys in both the thoracic RIP and preprocessed Abdo signals for each recording [24]. Respiratory peaks and valleys were identified at a 5 Hz sampling rate using an adaptive thresholding algorithm based on a sliding window (2 s). This window continuously computed a moving average baseline shifted by an adaptive safety margin of ±0.1 times the running SD to accommodate dynamic variations in breathing amplitude. Phase transitions were captured at the exact timestamps where the waveform intersected this adaptive baseline. Because these crossover points inherently occur shortly after a physical peak or valley, the algorithm executed a backward search over the completed phase using local extrema operators to pinpoint the precise coordinates. Finally, artifactual breaths were automatically excluded if their calculated amplitude fell below 30% of the preceding valid breath’s volume. A breath cycle was defined as the segment of the signal between 2 consecutive valleys and was considered invalid if it met at least one of the following criteria:

1. Recording time window: The breath occurs during the first 10 minutes and the last 2 minutes of the recording. This constraint aligns with the evaluation protocol of the AL system, which defines the valid analysis window starting 10 minutes after the beginning and ending 2 minutes before the end of the recording.
2. Baseline stability: The baseline change (the difference in amplitude) between 2 consecutive valleys is higher than 3 times the SD of all such valley-to-valley changes across the entire recording. This criterion filters out irregular or artifact-prone breaths due to baseline drift or motion.
3. Physiological breath interval: The interval between peaks of 2 breaths is less than 1.5 seconds, ensuring that only physiologically plausible breathing cycles are included and that spurious detections due to noise or nonrespiratory movements are excluded.

The synchronized signals were segmented into 1-minute intervals with a 5-second sliding step. One-minute intervals were selected to provide sufficient duration to capture the full dynamics of respiratory events, while the 5-second step allowed for nearly breath-level resolution across overlapping windows. The means of the rolling SD were calculated for each segment (mσ_acc_). A segment was excluded from analysis if it met any of the following conditions for either signal disconnection, an unrealistic breath count, or excessive body movement: (1) either the reference or Abdo signal was disconnected for more than 30% of the interval, (2) the number of valid breaths was greater than 30 or fewer than 2, or (3) the mσ_acc_ was greater than 3 times the median mσ_acc_ value within 15 consecutive segments. The adaptive thresholding of mσ_acc_ accounts for local acceleration variability trends, assuming that a typical 1-minute segment contains approximately 15 breaths. For each valid segment, the RR was computed as 60 divided by  *T*, where *T* is the average time in seconds between consecutive peaks of valid breaths.

### Performance Metrics

To evaluate performance on a discrete, breath-by-breath basis, a strict breath-matching tolerance protocol was enforced. A detected breath from the Abdo signal was classified as a true-positive match (*n*_correct_) if and only if its inspiration peak occurred within the temporal boundaries defined by 2 consecutive valleys of a single thoracic RIP respiratory cycle. Furthermore, to prevent ambiguous pairing or overcounting, instances where multiple or zero Abdo peaks fell within a single reference interval were classified as mismatched events, ensuring that each counted true positive corresponds precisely to the same physiological respiratory cycle. Breath detection performance was evaluated using standard classification metrics, including sensitivity (Se), positive predictive value (PPV), and *F*_1_-score, defined as follows:


$$Se=\frac{n_{correct}}{n_{ref}}$$

$$PPV=\frac{n_{correct}}{n_{test}}$$

$$F_{1}\text{-}score=\frac{2\times PPV\times Se}{PPV\text{+}Se}$$

where *n*_ref_ is the number of reference breaths identified in the thoracic RIP signal, *n*_test_ is the number of breaths detected in the Abdo signal, and *n*_correct_ is the number of correctly matched breaths between the 2 signals [13].

To evaluate the accuracy of RR estimation, the mean absolute percentage error (MAPE) was calculated as follows:


$$\text{MAPE}\text{=}\frac{1}{N}\sum_{i\text{=}1}^{N}\left|\frac{{\text{RR}}_{\text{ref},i}-{\text{RR}}_{\text{est},i}}{{\text{RR}}_{\text{ref},i}}\right|\times 100\text{\textbackslash{}\% }$$

where *N* represents the number of valid measurement segments, and RR_ref_ and RR_est_ denote the RRs derived from the reference thoracic RIP and the Abdo signals, respectively.

In addition, a Bland-Altman analysis was performed to assess agreement between the estimated and reference RR across the entire dataset. To quantify the reliability of RR estimation and understand how often estimation was possible, we calculated the coverage, defined as the proportion of 1-minute segments that were valid in both the RIP and Abdo signals relative to the total number of segments:


$$\text{Coverage (\textbackslash{}\%)}\text{=}\left(\frac{N_{\text{valid}}}{N_{\text{total}}}\right)\times 100$$

where *N*_valid_ is the number of 1-minute segments that successfully passed all quality exclusion criteria for both signals simultaneously, and *N*_total_ is the total number of 1-minute segments across the entire overnight recording time.

To assess waveform and trend similarity between the Abdo and thoracic RIP signals, we computed both distance correlation and mean squared error (MSE) across 1-minute sliding windows. Distance correlation was selected because it captures both linear and nonlinear relationships and is sensitive to differences in waveform shape [25,26]. Unlike Pearson correlation, it does not assume that a zero correlation implies independence, which is a critical distinction for physiological signals.

Accurate temporal alignment is essential for waveform similarity analysis because both distance correlation and MSE are sensitive to temporal offsets that may arise from sensor latency or body movement during sleep. Since synchronization performed only at the beginning of a recording cannot compensate for subsequent timing shifts, an automatic local alignment procedure was applied to each 1-minute window. Specifically, the optimal time lag was estimated by maximizing the cross-correlation between the Abdo and thoracic RIP signals within a constrained lag search range of ±2 s (±10 samples at 5 Hz), using a search step of 2 samples. This range is considered physiologically reasonable, as it falls within the duration of a typical respiratory cycle and ensures more robust waveform comparison under real-world sleep conditions. The Abdo signal was subsequently shifted by the estimated lag, and boundary samples affected by the temporal shift were discarded to avoid edge artifacts in the subsequent analysis. Prior to computing the final waveform similarity metrics, an explicit amplitude normalization protocol was executed: both the Abdo and thoracic RIP signals within each 1-minute window were *z*-score normalized (*z* = [*x −* µ] / σ) to center the baseline at µ=0 and fix the scaling parameter to unit variance (σ=1). This normalization eliminates arbitrary sensor scaling biases, ensuring that the metric evaluates underlying morphological and structural variations rather than scaling differences.

### Effects of Respiratory Event Types and Demographics of the Participants on Performance Metrics

To evaluate the effects of respiratory event types on monitoring performance, individual breath cycles and 1-minute signal segments were categorized into 4 mutually exclusive classes: N, H, OA, and CM (ie, CA and MA). Breath cycles were classified according to the timing of their inspiratory peaks. A breath was labeled as N if its inspiratory peak occurred outside any annotated respiratory event. Otherwise, it was assigned the label corresponding to the annotated event encompassing its inspiratory peak (H, OA, or CM). One-minute signal segments were classified using the same 4 categories. Segments containing no respiratory events were labeled as N, whereas segments containing one or more respiratory events were assigned the event type occupying the greatest cumulative duration within the 1-minute interval.

To assess the influence of respiratory event type on breath detection and RR estimation, participant-level performance metrics, including Se, PPV, *F*_1_-score, MAPE, limits of agreement (LOA), and coverage, were first calculated separately for each event category within each participant. Linear mixed-effects models were then fitted using respiratory event type as a fixed effect and participant identity as a random intercept to account for within-participant correlation [27]. The effects of participant demographics on whole-recording performance metrics were evaluated using 4 categorical variables: AHI (<5, 5.0‐14.9, and >14.9 events/h), ODI (<5, 5.0‐14.9, and >14.9 events/h), BMI (<25, 25.0‐29.9, and >29.9 kg/m²), and sex (male or female participants). Each demographic factor was analyzed independently. Differences among the 3-level groups were assessed using the Kruskal-Wallis test followed by Dunn-Šidák post hoc pairwise comparisons, whereas differences between sexes were evaluated using the 2-tailed Mann-Whitney *U* test.

To investigate the effects of respiratory event type and participant demographics on waveform similarity, distance correlation and MSE were analyzed using the original 1-minute segment-level data. Because each participant contributed multiple repeated observations, segment-level linear mixed-effects models were fit directly to the unaggregated dataset. Respiratory event type or a single demographic factor was included as the fixed effect in separate models, while participant identity was modeled as a random intercept to account for within-participant dependence. Post hoc pairwise comparisons were performed using Wald tests on the fixed-effects contrasts (coefTest, MATLAB). All statistical analyses were performed using the Statistics and Machine Learning Toolbox in MATLAB (MathWorks), and statistical significance was defined as *P*<.05.

### Ethical Considerations

This study was conducted in accordance with the principles of the Declaration of Helsinki. Approval was granted by the institutional review board of UNIST (UNISTIRB-24‐006-A). Written informed consent was obtained from all participants. No identifiable personal data or images of participants are included in this manuscript.

## Results

**Figure 5.**
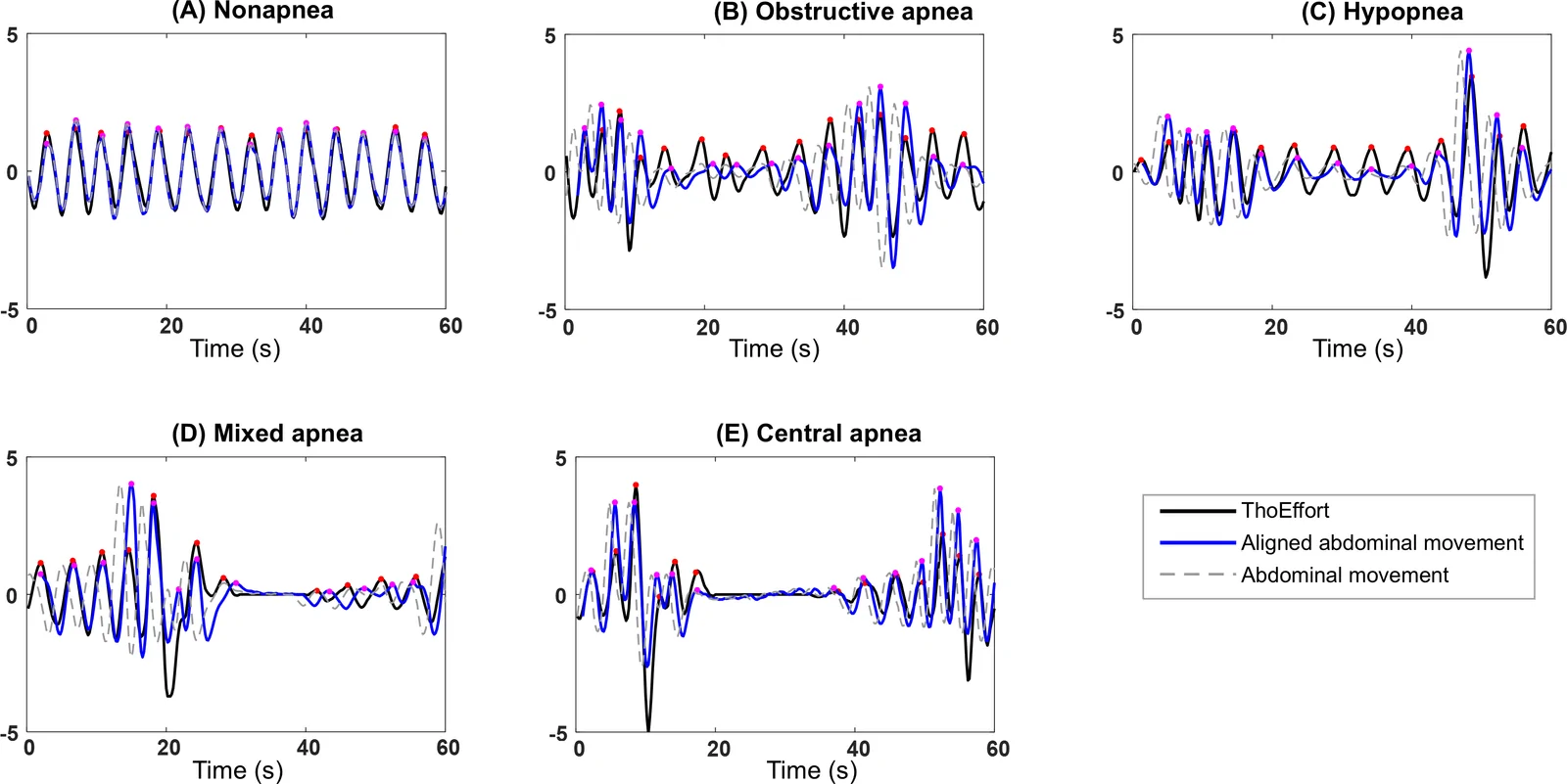
Examples of thoracic respiratory inductance plethysmography (RIP) and abdominal movement (Abdo) signals measured during different respiratory event types: (A) nonapnea, (B) obstructive apnea, (C) hypopnea, (D) mixed apnea, and (E) central apnea. The solid black, solid blue, and dashed gray lines represent the thoracic RIP (ThoEffort), the time-aligned Abdo signal, and the preprocessed Abdo signal, respectively. The detected peaks in ThoEffort and in the time-aligned Abdo signal are marked in red and magenta colors, respectively.

Of the 37 recordings collected, 3 were excluded because of technical failures during unconstrained home monitoring. Two recordings were excluded due to Soomirang system issues, including one with missing accelerometer data and another with critical sampling-time errors. The third recording was excluded because of a hardware-related connection failure of the thoracic RIP belt, which resulted in prolonged signal flatlining and high-amplitude contact jitter caused by sensor detachment or cable disconnection. All exclusions were due to technical issues and were unrelated to participant characteristics or clinical outcomes. Thirty-four recordings were used for analysis; 8 recordings were obtained from female participants and 26 recordings from male participants. The distributions of participants across different obstructive sleep apnea (OSA) severity levels of normal, mild, moderate, and severe were 14, 9, 1, and 10, respectively. Figure 5 presents representative examples of breath-cycle detection in thoracic RIP and Abdo signals after preprocessing and normalization across segments of different respiratory event types.

The Abdo signal shows good performance in breath detection, with overall sensitivity, PPV, and *F*_1_-score of 82.44% (69.58%‐97.25%), 76.22% (50.06%‐92.85%), and 78.99% (60.27%‐94.16%), respectively; and in RR estimation, with a MAPE of 5.42% (2.04%‐12.98%), a Bland-Altman bias of 0.21 breaths per minute (bpm), and a Bland-Altman LOA of ±3 bpm (−2.8 to 3.2 bpm). The RR coverage was high at 84.74% (27.82%‐95.8%). For pattern similarity comparison, the distance correlation and MSE between the Abdo and reference RIP signals were moderate at 0.73 (0.51‐0.92) and 0.64 (0.25‐1.78), respectively.

The number of valid breaths in the reference/Abdo signal across respiratory event categories was 131,778/132,503 for N, 8581/7849 for H, 16,191/15,993 for OA, and 1055/2139 for CM, respectively. The number of valid segments across respiratory event categories was 8445 for N, 1239 for H, 2140 for OA, and 329 for CM. Table 2 summarizes the impact of different respiratory event types on the performance of breath detection and RR estimation. The Se and *F*_1_-score were highest during N segments and showed a gradual decline across H and OA, with the lowest values observed in CM segments. Specifically, sensitivity decreased from 83.17% in N segments to 70.81% in CM segments (*P*<.001). A similar pattern was observed for *F*_1_-score, which dropped from 82.95% in N segments to 46.78% in CM segments (*P*<.001). While PPV was relatively high in H and OA segments (86.27% and 75.25%, respectively), it dropped substantially to 34.92% in CM segments (*P*<.05). Overall, all performance metrics showed a marked reduction from N to CM segments (*P*<.001), indicating decreased breath-detection reliability in the presence of more complex or irregular respiratory events. The RR estimation performance gradually degraded with event severity, as indicated by a significant overall effect of event type on MAPE and LOA (*P*<.001). The lowest error occurred during N segments (MAPE=4.72%; LOA=−2.6 to 3.0 bpm) and increased progressively across H (MAPE=6.15%; LOA=−3.0 to 3.2 bpm) and OA (MAPE=7.31%; LOA=−2.9 to 3.6 bpm) events. The error peaked during CM segments (MAPE=9.72%; LOA=−4.8 to 4.8 bpm), with post hoc testing revealing that this CM group had a significantly higher MAPE than all other categories (*P*<.001 for all pairwise comparisons). Conversely, variations in MAPE among the N, H, and OA groups were not statistically significant. Regarding data coverage, the highest values were observed during OA (92.34%) and H (88.54%) segments, followed by N segments (81.93%), with coverage dropping sharply to a minimum during CM segments (57.45%). Pairwise comparisons for patient-level coverage showed that N segments differed significantly from H (*P*=.002), OA (*P*<.001), and CM (*P*=.02). Coverage also differed significantly between OA and CM (*P*=.04), whereas the remaining differences between H and OA (*P*=.06) or CM (*P*=.63) did not reach statistical significance.

**Table 2.** Effect of respiratory events on breath detection and respiratory rate estimation performance metrics.

Performance metrics	N^a^	H^b^	OA^c^	CM^d^
Se (%)^e^	83.17	78.91	74.35	70.81
PPV (%)^f^	82.72	86.27	75.25	34.92
*F*_1_-score (%)	82.95	82.42	74.78	46.78
MAPE (%)^g^	4.72	6.15	7.31	9.72
LOA^h^ (bpm^i^)	2.8 (−2.6 to 3.0)	3.1 (−3 to 3.2)	3.3 (−2.9 to 3.6)	4.8 (−4.8 to 4.8)
Coverage (%)	81.93	88.54	92.34	57.45

a N: nonapnea.

b H: hypopnea.

c OA: obstructive apnea.

d CM: central apnea and mixed apnea.

e Se: sensitivity.

f PPV: positive predictive value.

g MAPE: mean absolute percentage error.

h LOA: limits of agreement.

i bpm: breaths per minute.

**Figure 6.**
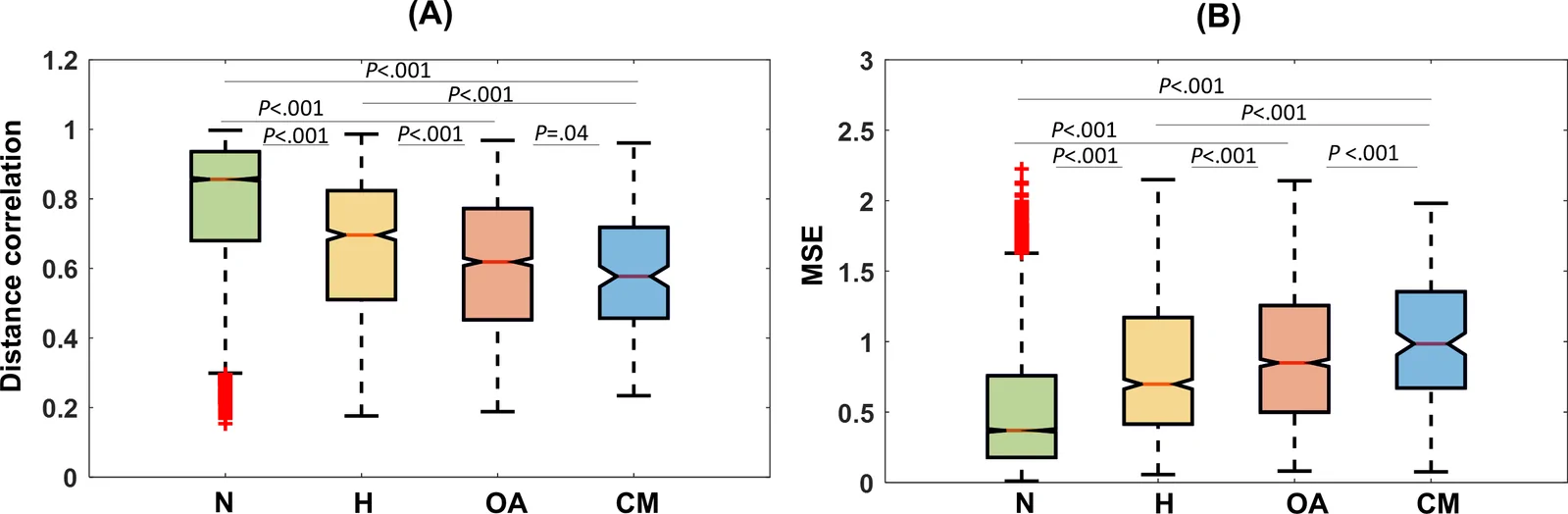
Effect of respiratory events on pattern similarity performance indices: distance correlation and mean squared error (MSE). CM: central apnea and mixed apnea; H: hypopnea; N: nonapnea; OA: obstructive apnea. Statistical significance was defined as *P*<.05.

Figure 6 and Table S1 in Multimedia Appendix 1 present the effects of different respiratory event types on the segment-level pattern similarity metrics. The highest distance correlation was observed in N segments (0.78±0.192), indicating the strongest pattern similarity and waveform alignment between the Abdo and thoracic RIP signals. Conversely, the lowest distance correlation occurred in CM segments (0.59±0.17), reflecting degraded morphological agreement during mixed or central apneas. The distance correlation in H segments (0.66±0.19) was significantly higher than that observed in both OA and CM segments (*P*<.001). Furthermore, OA segments (0.61±0.17) exhibited a slightly higher distance correlation than CM segments (*P*=.04). In terms of MSE, the lowest MSE values were achieved during N segments (0.53±0.45), indicating the greatest overall agreement. The highest MSE occurred in CM segments (1.01±0.43), suggesting the largest deviation in waveform shape. The MSE in H segments (0.8±0.47) was significantly lower than that in OA and CM segments (*P*<.001). Additionally, OA segments (0.88±0.45) yielded a significantly lower MSE than CM segments (*P*<.001).

Table 3 summarizes the impacts of AHI, ODI, BMI, and biological sex on breath detection and RR estimation performance. Se, PPV, and *F*_1_-score noticeably declined as AHI, ODI, and BMI increased. All 3 performance metrics were consistently higher in female participants than in male participants. Statistically significant variations in Se were observed across AHI groups (*P*=.01), ODI groups (*P*=.004), and between sexes (*P*=.04), whereas variations across BMI groups were nonsignificant (*P*=.42). For PPV, no significant differences were found across AHI, ODI, or BMI groups, but a statistically significant difference was observed between male and female cohorts (*P*=.049). *F*_1_-score variations did not reach statistical significance across AHI or BMI groups, but significant differences were found across ODI groups (*P*=.048) and between female and male participants (*P*=.049). For RR estimation performance, MAPE and LOA generally increased with higher AHI, ODI, and BMI. Performance metrics were consistently lower in female compared to male participants. Statistically significant differences in MAPE were observed across AHI groups (*P*=.02) and ODI groups (*P*<.001). However, variations in MAPE across BMI groups (*P*=.57) and between sexes (*P*=.38) did not reach statistical significance. Regarding data coverage, no significant differences were observed across any of the evaluated demographic groups.

**Figure 7.**
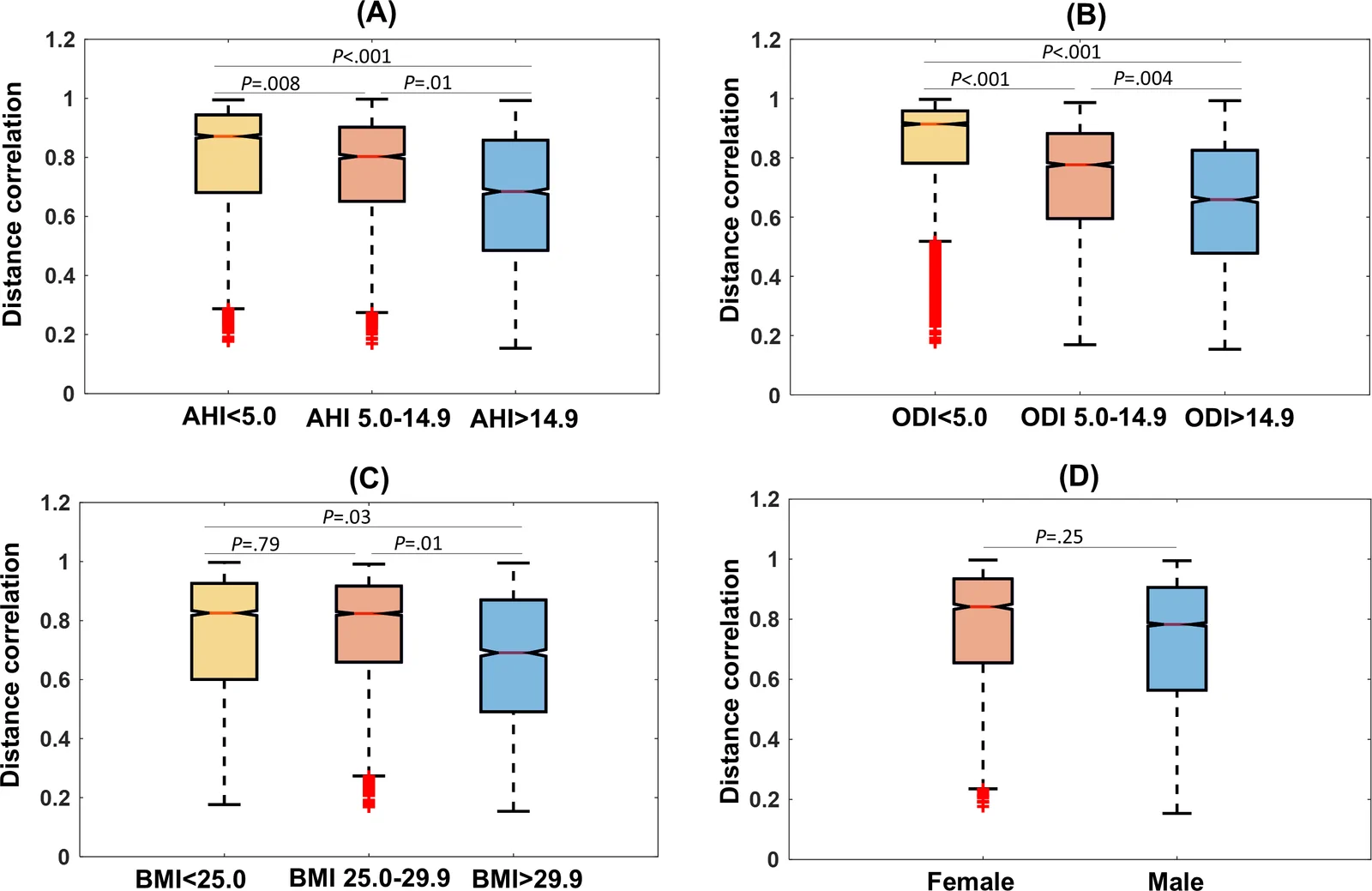
Influences of the apnea-hypopnea index (AHI), oxygen desaturation index (ODI), BMI, and sex on the distance correlation metric of pattern similarity. Statistical significance was defined as *P*<.05.

**Figure 8.**
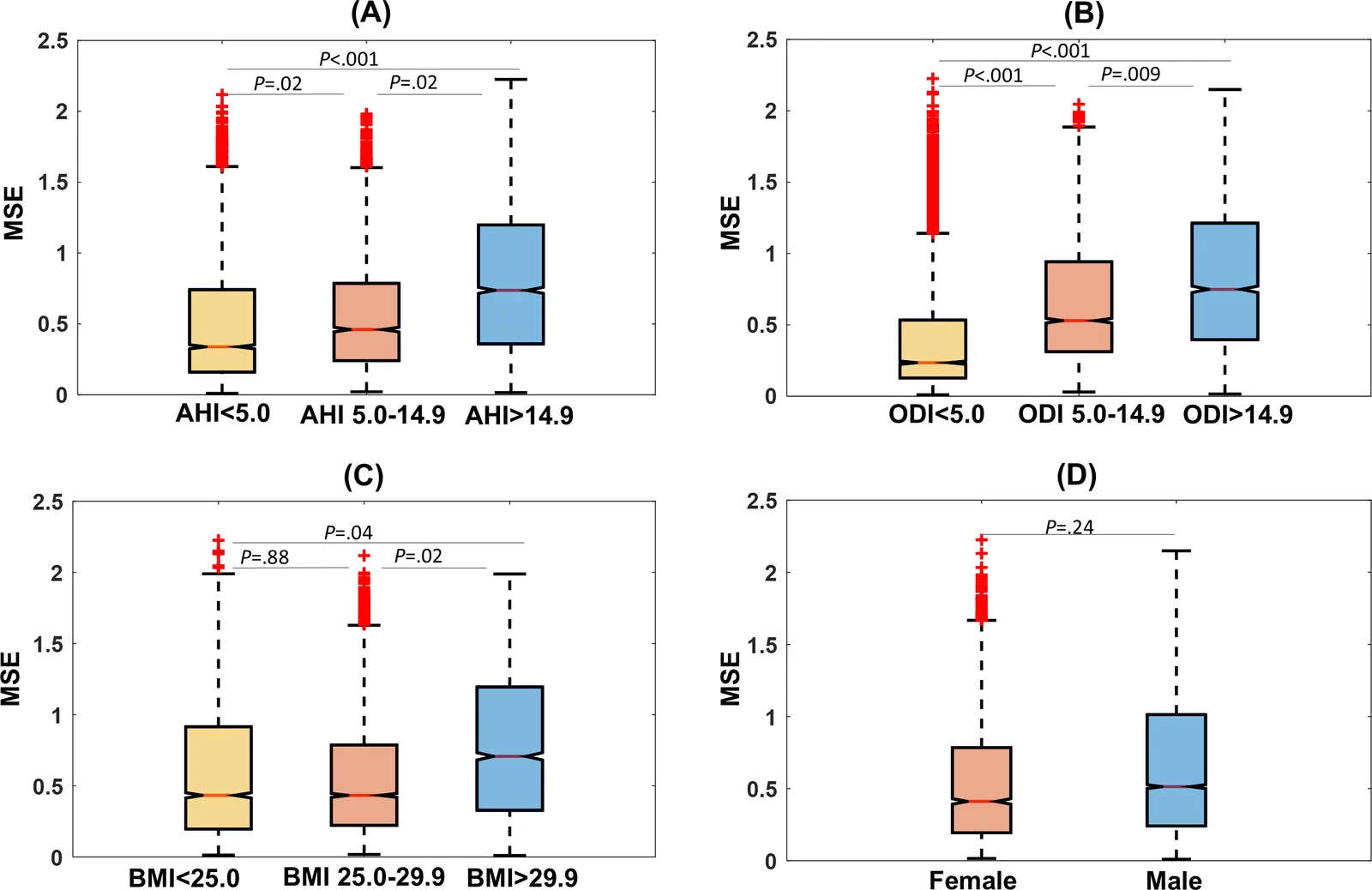
Influences of the apnea-hypopnea index (AHI), oxygen desaturation index (ODI), BMI, and sex on the mean squared error (MSE) of pattern similarity. Statistical significance was defined as *P*<.05.

Figure 7, Figure 8, and Table S2 in Multimedia Appendix 1 present the effects of AHI, ODI, BMI, and sex on distance correlation and MSE. A significant difference in both metrics was observed across the AHI groups. The highest distance correlation and lowest MSE were observed in participants with normal sleep profiles (AHI<5 events/h; 0.8±0.19 and 0.48±0.45, respectively), whereas participants with moderate-to-severe SA (AHI>14.9 events/h) exhibited the lowest distance correlation (0.65±0.2) and highest MSE (0.81±0.48). Pairwise comparisons revealed significant differences between all AHI cohorts for both distance correlation (normal vs mild: *P*=.008; normal vs moderate-to-severe: *P*<.001; mild vs moderate-to-severe: *P*=.01) and MSE (normal vs mild: *P*=.02; normal vs moderate-to-severe: *P*<.001; mild vs moderate-to-severe: *P*=.02). An identical pattern was observed across ODI categories, with all pairwise differences for both metrics reaching high statistical significance (*P*<.009). For BMI, participants with a normal weight (BMI<25 kg/m^2^) and overweight participants (BMI=25–29.9 kg/m^2^) showed no significant differences in waveform similarity (*P*=.79 for distance correlation; *P*=.88 for MSE). However, waveform agreement deteriorated in participants with obesity (BMI>29.9 kg/m²), resulting in the lowest overall distance correlation (0.67±0.21) and the highest MSE (0.77±0.51). Obese participants showed significantly lower distance correlation and higher MSE compared to both normal weight (distance correlation: *P*=.03; MSE: *P*=.03) and overweight cohorts (distance correlation: *P*=.01; MSE: *P*=.02). Lastly, no statistically significant sex-based differences were observed for both pattern similarity metrics.

**Table 3.** Effects of apnea-hypopnea index (AHI), oxygen desaturation index (ODI), BMI, and sex on breath detection and respiratory rate estimation performance indices.

Performance indices	AHI			ODI			BMI			Sex	
	<5	5.0‐14.9	>14.9	<5	5.0‐14.9	>14.9	<25	25.0‐29.9	>29.9	Female	Male
Se^a^ (%)	85.52	82.83	78.2	86.77	81.68	77.49	84.16	83.43	77.5	86.27	80.6
PPV^b^ (%)	77.98	77.4	68.94	78.42	77.16	68.94	77.38	75.78	70.63	81.25	72.9
*F*_1_-score (%)	81.33	79.93	72.97	82.38	79.35	72.97	80.63	79.42	73.91	83.69	76.6
MAPE^c^ (%)	4.09	5.87	6.75	3.28	6.16	6.75	5.1	4.93	6.56	4.53	5.69
LOA^d^ (bpm)	2.6 (−2.4 to 2.7)	3.1 (−2.8 to 3.4)	3.3 (−3.1 to 3.5)	2.0 (−1.9 to 2.1)	3.3 (−3.0 to 3.6)	3.3 (−3.1 to 3.5)	2.8 (−2.6 to 3.0)	2.7 (−2.5 to 2.9)	3.5 (−3.2 to 3.7)	2.7 (−2.6 to 2.8)	3 (−2.8 to 3.3)
Cov^e^ (%)	84.31	91.48	79.77	82.91	90.97	79.77	86.93	81.98	82.25	87.17	83.99

a Se: sensitivity.

b PPV: positive predictive value.

c MAPE: mean absolute percentage error.

d LOA: limits of agreement.

e Cov: coverage.

## Discussion

This study presents a characterization of the respiratory signals recorded by a wireless, abdomen-worn sensor during a full night of HSAT. The measured Abdo signal was compared with the respiratory signal recorded by the thoracic RIP belt in terms of breath-cycle detection, RR estimation, and waveform morphology. Additionally, the influences of sleep and demographic factors on performance metrics were investigated.

The Abdo signal demonstrated moderate performance for respiratory measurement across several key metrics. For breath-cycle detection, it achieved a sensitivity of 82.44% (range: 69.58‐97.25), a PPV of 76.22% (range: 50.06‐92.85), and an *F*_1_-score of 78.99% (range: 60.27‐94.16). In RR estimation, the MAPE was 5.42%, with an average LOA of ±3 bpm and coverage of 84.74%. For waveform similarity, the average distance correlation was 0.73, and the MSE was 0.64. These results suggest the Abdo signal is moderately similar to the reference signal, and that the abdomen-worn sensor offers a promising approach for noninvasive respiratory monitoring during sleep. Comparable studies support these findings. Cerina et al evaluated suprasternal pressure signals for breath detection against thoracic RIP and reported a sensitivity of 78.21%, a PPV of 78.04%, and an *F*_1_-score of 77.62%. For RR estimation, their system achieved a MAPE of 4.14%, an average LOA of ±2.78 bpm, and coverage of 84.26% when compared with the thoracic RIP signal. Their evaluation of waveform similarity, based on breath amplitude, yielded an average distance correlation of 0.692 [13]. Schipper et al [17] used a deep learning model to estimate respiratory effort from accelerometer data and reported a sensitivity and PPV of 93.7% and 93.5%, respectively, for breath-cycle detection. Their morphology similarity assessment showed an average Pearson correlation coefficient of 0.83 and an MSE of 0.33. While these comparisons offer useful context, direct performance comparisons are limited by differences in study populations and methodologies. For instance, the dataset used in the study of Schipper et al [17] included fewer respiratory events, which may have contributed to higher detection performance. Additionally, their use of Pearson correlation assumes linear relationships and independence of data points, which might not fully capture the complex, potentially nonlinear and temporally dependent nature of physiological signals recorded during sleep. In contrast, our use of distance correlation allows for a more general assessment of waveform similarity. Cerina et al [13] compared respiratory pattern features across sequences of valid breaths. While this approach offers valuable insights, its robustness may be limited when valid breaths occur at different times in the reference and test signals. In such cases, sequence-based comparisons could be affected by temporal misalignment, potentially leading to less accurate assessments.

The performance of the abdomen-worn sensor for respiratory signal detection is strongly influenced by the factor of respiratory event types. The metrics of breath-cycle detection and RR estimation are considerably higher in N and H events compared to OA and CM events. There is a significantly higher distance correlation and a lower MSE in N and H events compared to OA and CM events. The results indicate that the respiratory signal detection of the abdomen-worn sensor worsens as the severity of the respiratory event increases. This observation is similar to those observed in signals measured from a microphone and an accelerometer, where the quality of the respiratory signal measured from these devices reduced significantly in severe respiratory events [13,15,16]. One possible explanation is that irregular breathing patterns complicate the relationship between thoracic and Abdo signals and overall body circumference, making accurate signal tracking more challenging [28]. In abnormal breathing scenarios like paradoxical breathing, thoracic and abdominal signals may become out of phase, further reducing signal agreement [28,29]. Moreover, since the abdomen-worn sensor captures Abdo at a single location, it may not fully reflect overall Abdo and can be more susceptible to motion artifacts. The presence of noise during breathing-disordered events contributes to a reduced distance correlation, increased MSE, and greater differences in RR between Abdo and thoracic RIP signals. Although respiratory event type influenced both breath detection and RR estimation, the abdomen-worn sensor maintained moderate performance during OA and CM, achieving a breath detection sensitivity greater than 70%, a MAPE below 10%, and LOA within ±4.8 bpm.

The performance of the abdomen-worn sensor was compared across participant groups stratified by AHI, ODI, BMI, and sex. The breath-cycle detection and RR estimation performance declined with increasing AHI and ODI. Significant differences in distance correlation and MSE were observed between groups with varying AHI and ODI levels, likely due to the higher frequency of respiratory events in participants with more severe OSA. These respiratory events introduce irregularities that affect signal quality and detection accuracy. Analysis by sex revealed that while waveform similarity metrics showed no significant intergroup differences, breath-cycle detection performance was slightly elevated in the female cohort compared with the male cohort. Attributing this discrepancy solely to sex remains challenging due to a pronounced imbalance in baseline clinical severity; the average AHI and ODI were substantially lower among female participants (7.60 and 7.13 events/h) than among male participants (27.32 and 22.71 events/h). This heavily skewed distribution in baseline apnea severity likely confounded the performance differences observed between sexes. BMI was another factor influencing the performance metrics. Participants with a BMI>29.9 showed significantly lower distance correlation and higher MSE compared to those with a BMI<25 or between 25.0 and 29.9. This result suggests that higher body weight may reduce the accuracy of respiratory signal detection in both the RIP belt and the abdomen-worn sensor. The added mass around the chest and abdomen may also reduce the strength or clarity of the respiratory signal. These observations are consistent with existing literature, reinforcing the impact of body composition on respiratory monitoring accuracy using wearable sensors [30,31]. However, it is also noted that in our population, the participants with BMI>29.9 have higher AHI compared to the people with lower BMI; therefore, the significant difference between these groups could be influenced by respiratory events. Beyond these clinical and demographic factors, part of the reduced agreement observed across participant subgroups may stem from the different anatomical measurement sites. The abdomen-worn sensor captures abdominal movement, whereas the reference RIP belt records thoracic respiratory motion. Because the thorax and abdomen function as distinct respiratory compartments, their movements can naturally differ in amplitude or become temporarily asynchronous during altered breathing, particularly during respiratory events. Consequently, some degree of disagreement between the 2 signals is physiologically expected and may contribute to the observed reduction in waveform agreement.

While the baseline performance metrics reported here demonstrate a stable foundation for tracking abdominal movements, evaluating downstream clinical diagnostic capacities remains outside the scope of this specific work. The underlying characteristics evaluated in this study provide the necessary metrological foundation that allows these raw signals to be viably used in deep learning applications. Indeed, our previous work has successfully demonstrated that once these fundamental respiratory dynamics are established, they can be integrated into advanced AI frameworks for automated SA screening, AHI estimation, and SA severity classification [18].

This study has several limitations. First, although abnormal breathing events were shown to affect the Abdo signal, the influence of other factors such as sleep position, sleep stage, and snoring could not be evaluated due to the lack of corresponding data in the reference HSAT system [17]. Second, the reference signal was obtained from a thoracic RIP belt rather than an abdominal belt, as dictated by the standard configuration of the AL system. As discussed, chest and abdominal movements may diverge during disordered breathing; thus, this measurement location mismatch represents a practical limitation when directly comparing thoracic and abdominal waveforms. Third, although conducting the study in a home setting allows participants to sleep in a familiar environment, providing more natural usage conditions for wearable devices, a more thorough evaluation of the abdomen-worn sensor should be performed in a controlled sleep lab using PSG as the gold standard. Fourth, the number of CM events was relatively small compared to other respiratory event types, limiting the ability to draw statistically robust conclusions regarding signal behavior during CM events. Fifth, the number of participants was relatively small, and there was a sex imbalance, which makes it harder to fully understand how demographic factors affect performance. Despite these limitations, to the best of our knowledge, this is the first study to analyze Abdo signals from a wireless, single-point abdomen-worn sensor for respiratory movement detection, highlighting its potential as a simple and practical tool for monitoring thoracoabdominal movement during sleep.

This study characterized the Abdo signal measured during HSAT using a simple, wireless, abdomen-worn sensor. We assessed the performance of the Abdo signal in breath-cycle detection, RR estimation, and waveform similarity, using thoracic RIP from an HSAT device as the reference. Additionally, we analyzed the influence of sleep-related and demographic factors on the signal quality and detection performance. The Abdo signal demonstrated moderate agreement with the reference across both breath-by-breath detection and waveform morphology metrics. However, this agreement was significantly influenced by factors such as apnea severity and BMI. These findings support the potential of a small, wireless, abdomen-worn sensor for home-based respiratory monitoring. Future studies should aim to enhance performance in sleep laboratory settings and explore its application in OSA and central sleep apnea (CSA) classification.

## Supplementary material

10.2196/93351 Multimedia Appendix 1 Effects of respiratory event types and participant characteristics on pattern similarity performance metrics.

10.2196/93351 Multimedia Appendix 2 AI prompt transcripts.

## Abbreviations

Abdo: abdominal movement
AHI: apnea-hypopnea index
AL: ApneaLink Air
CSA: central sleep apnea
HSAT: home sleep apnea test
LOA: limits of agreement
MAPE: mean absolute percentage error
MSE: mean squared error
ODI: oxygen desaturation index
OSA: obstructive sleep apnea
PPV: positive predictive value
PSG: polysomnography
RIP: respiratory inductance plethysmography
RR: respiratory rate
SA: sleep apnea

## References

[1] Cowie MR, Linz D, Redline S, Somers VK, Simonds AK (2021). Sleep disordered breathing and cardiovascular disease: JACC state-of-the-art review. J Am Coll Cardiol.

[2] Yeghiazarians Y, Jneid H, Tietjens JR (2021). Obstructive sleep apnea and cardiovascular disease: a scientific statement from the American Heart Association. Circulation.

[3] Jean-Louis G, Zizi F, Clark LT, Brown CD, McFarlane SI (2008). Obstructive sleep apnea and cardiovascular disease: role of the metabolic syndrome and its components. J Clin Sleep Med.

[4] Hussein O, Alkhader A, Gohar A, Bhat A (2024). Home sleep apnea testing for obstructive sleep apnea. Mo Med.

[5] Kapur VK, Auckley DH, Chowdhuri S (2017). Clinical practice guideline for diagnostic testing for adult obstructive sleep apnea: an American Academy of Sleep Medicine clinical practice guideline. J Clin Sleep Med.

[6] Orr JE, Ayappa I, Eckert DJ (2021). Research priorities for patients with heart failure and central sleep apnea. An official American Thoracic Society research statement. Am J Respir Crit Care Med.

[7] Berry RB, Brooks R, Gamaldo C (2017). AASM scoring manual updates for 2017 (version 2.4). J Clin Sleep Med.

[8] Lymberis A, Rossi D (2004). Wearable Ehealth Systems for Personalised Health Management: State of the Art and Future Challenges.

[9] Rahman MS, Chowdhury S, Rasheduzzaman M, Doulah ABMSU (2024). Artificial intelligence-based algorithms and healthcare applications of respiratory inductance plethysmography: a systematic review. Algorithms.

[10] Coronel C, Wiesmeyr C, Garn H (2019). Measurement of respiratory effort in sleep by 3D camera and respiratory inductance plethysmography. Somnologie.

[11] Dong S, Wen L, Li Y (2024). Remote respiratory variables tracking with biomedical radar-based IoT system during sleep. IEEE Internet Things J.

[12] Massaroni C, Nicolò A, Lo Presti D, Sacchetti M, Silvestri S, Schena E (2019). Contact-based methods for measuring respiratory rate. Sensors (Basel).

[13] Cerina L, Papini GB, Fonseca P (2024). Quantitative validation of the suprasternal pressure signal to assess respiratory effort during sleep. Physiol Meas.

[14] Glos M, Sabil A, Jelavic KS, Schöbel C, Fietze I, Penzel T (2018). Characterization of respiratory events in obstructive sleep apnea using suprasternal pressure monitoring. J Clin Sleep Med.

[15] Montazeri Ghahjaverestan N, Fan W, Aguiar C, Yu J, Bradley TD (2022). Respiratory motion and airflow estimation during sleep using tracheal movement and sound. Nat Sci Sleep.

[16] Ryser F, Hanassab S, Lambercy O, Werth E, Gassert R (2022). Respiratory analysis during sleep using a chest-worn accelerometer: a machine learning approach. Biomed Signal Process Control.

[17] Schipper F, Grassi A, Overeem S, Fonseca P (2023). A deep-learning approach to assess respiratory effort with a chest-worn accelerometer during sleep. Biomed Signal Process Control.

[18] Dang TH, Kim SM, Choi MS, Hwan SN, Min HK, Bien F (2025). An automated algorithm for obstructive sleep apnea detection using a wireless abdomen-worn sensor. Sensors (Basel).

[19] Kim S, Dang TH, Cho H (2025). Clinical study of an integrated sensor system for detection and classification of obstructive sleep apnea (OSA). Sci Rep.

[20] Niu H, Li H, Li N (2024). Fringing‐effect‐based capacitive proximity sensors. Adv Funct Materials.

[21] Bhattacharjee R, Benjafield A, Blase A (2021). The accuracy of a portable sleep monitor to diagnose obstructive sleep apnea in adolescent patients. J Clin Sleep Med.

[22] Dzięciołowska-Baran E, Gawlikowska-Sroka A, Szczurowski J (2020). Medical Research and Development.

[23] Gallagher NB (2020). Savitzky-Golay smoothing and differentiation filter. https://www.scribd.com/document/902695337/SavitzkyGolay.

[24] Dang TH, Jang GY, Lee K, Oh TI (2023). Motion artifacts reduction for noninvasive hemodynamic monitoring of conscious patients using electrical impedance tomography: a preliminary study. Sensors (Basel).

[25] Székely GJ, Rizzo ML, Bakirov NK (2007). Measuring and testing dependence by correlation of distances. Ann Statist.

[26] Edelmann D, Móri TF, Székely GJ (2021). On relationships between the Pearson and the distance correlation coefficients. Stat Probab Lett.

[27] Brown VA (2021). An introduction to linear mixed-effects modeling in R. Adv Methods Pract Psychol Sci.

[28] Sayas J, Lalmolda C, Corral M (2024). Measurement of thoraco-abdominal synchrony using respiratory inductance plethysmography: technical aspects and a proposal to overcome its limitations. Expert Rev Respir Med.

[29] Denton E, Bondarenko J, Hew M (2022). Complex Breathlessness (ERS Monograph).

[30] Roos LG, Slavich GM (2023). Wearable technologies for health research: opportunities, limitations, and practical and conceptual considerations. Brain Behav Immun.

[31] Montazeri K, Jonsson SA, Agustsson JS, Serwatko M, Gislason T, Arnardottir ES (2021). The design of RIP belts impacts the reliability and quality of the measured respiratory signals. Sleep Breath.

