# Supplementary material for: Characteristics of Abdominal Movement Signals Measured by a Wireless Abdomen-Worn Sensor During Home Sleep Apnea Testing: Pilot Observational Study
Source: JMIR Form Res. 2026 Sep 1;10:e93351. doi: 10.2196/93351 (PMC13532689; doi:10.2196/93351)
Supplement: Supplementary file 1 [file formative-v10-e93351-s001.docx]

# **Multimedia Appendix 1**

**Table S1** Effect of respiratory events on patterns similarity performance indexes. MSE: mean square error, N: non-apnea, H: hypopnea, OA: obstructive apnea, and CM: central apnea and mixed apnea

| Metrics | N | H | OA | CM |
| --- | --- | --- | --- | --- |
| Distance correlation | 0.785 $\pm$ 0.192 | 0.66 $\pm$ 0.19 | 0.61 $\pm$ 0.17 | 0.59 $\pm$0.17 |
| MSE | 0.53 $\pm$ 0.45 | 0.80 $\pm$0.47 | 0.88 $\pm$0.45 | 1.01 $\pm$0.43 |

**Table S2** Effects of apnea hypopnea index (AHI), oxygen desaturation index (ODI), body mass index (BMI), and sex on performance metrics of pattern similarity. Dcor: distance correlation, MSE: mean square error

|  | AHI | | | ODI | | | BMI | | | Sex | | |
| --- | --- | --- | --- | --- | --- | --- | --- | --- | --- | --- | --- | --- |
|  | < 5 | 5 – 14.9 | > 14.9 | < 5 | 5 – 14.9 | > 14.9 | < 25 | 25 – 29.9 | >29.9 | F | M |  |
| Dcor | 0.81  $\pm$  0.19 | 0.73  $\pm$  0.19 | 0.65  $\pm$  0.2 | 0.84  $\pm$  0.18 | 0.72  $\pm$  0.19 | 0.65  $\pm$  0.2 | 0.75  $\pm$ 0.21 | 0.77  $\pm$ 0.19 | 0.67  $\pm$  0.21 | 0.78  $\pm$ 0.19 | 0.73 $\pm$  0.21 |  |
| MSE | 0.48  $\pm$  0.45 | 0.65  $\pm$  0.44 | 0.81  $\pm$  0.48 | 0.41  $\pm$  0.43 | 0.66  $\pm$  0.44 | 0.81  $\pm$  0.48 | 0.59  $\pm$ 0.48 | 0.56  $\pm$ 0.44 | 0.77  $\pm$ 0.51 | 0.54  $\pm$ 0.44 | 0.65$\pm$  0.49 |  |
