# Supplementary material for: Characteristics of Abdominal Movement Signals Measured by a Wireless Abdomen-Worn Sensor During Home Sleep Apnea Testing: Pilot Observational Study
Source: JMIR Form Res. 2026 Sep 1;10:e93351. doi: 10.2196/93351 (PMC13532689; doi:10.2196/93351)
Supplement: Supplementary file 2 [file formative-v10-e93351-s002.docx]

# **Multimedia Appendix 2: AI Prompt Transcripts**

1. **ChatGPT-5.5**

***User Prompt:*** Refine this paragraph [While the baseline performance metrics reported here demonstrate a stable foundation for tracking abdominal movements, evaluating downstream clinical diagnostic capacities remains outside the scope of this specific work. The underlying characteristics evaluated in this study provide the necessary metrological foundation that allows these raw signals to be reliably utilized in deep learning applications. Indeed, our previous work has successfully demonstrated that once these fundamental respiratory dynamics are established, they can be integrated into advanced AI frameworks for automated SA screening, AHI estimation, and SA severity classification [18].]

***AI response:***

Here's a more polished and natural version that reads well in a Discussion section:

While the baseline performance metrics reported in this study demonstrate the feasibility of reliably tracking abdominal respiratory movements, evaluating downstream clinical diagnostic performance is beyond the scope of the present work. Instead, this study establishes the sensor's fundamental measurement performance by characterizing its respiratory waveform fidelity and breath-tracking capabilities. These findings provide the metrological foundation for the reliable use of the Abdo signal in downstream deep learning applications. Building upon this foundation, our previous work demonstrated that these validated respiratory signals can be effectively integrated into an AI-based framework for automated sleep apnea screening, AHI estimation, and sleep apnea severity classification [18].

Why this version is stronger

- "Clinical diagnostic performance" is more standard than "clinical diagnostic capacities."
- "Establishes the sensor's fundamental measurement performance" clearly states the purpose of the current study.
- "Respiratory waveform fidelity and breath-tracking capabilities" reminds readers what was actually evaluated.
- "Building upon this foundation" creates a smoother transition to your previous work.
- "Can be effectively integrated" is more measured than "has successfully demonstrated that... can be integrated," avoiding unnecessary emphasis while still making the connection between the two studies.

Overall, this version presents the engineering validation as a natural prerequisite for the clinical AI framework, which aligns well with the narrative you've been developing throughout the manuscript.

***User Prompt:***

I do not like the word "reliable use", can you change a bit? because with moderate performance, saying reliable is a bit strong.

***AI response:***

I agree. With an F1-score around 79% and moderate waveform agreement, the phrase "reliable use" may sound stronger than the evidence supports. It is better to use more measured language such as "supports," "facilitates," "enables," or "serves as a foundation for."

Here are a few alternatives.

Option 1 (Recommended)

While the baseline performance metrics reported in this study demonstrate the feasibility of tracking abdominal respiratory movements, evaluating downstream clinical diagnostic performance is beyond the scope of the present work. Instead, this study establishes the sensor's fundamental measurement performance by characterizing its respiratory waveform fidelity and breath-tracking capabilities. These findings provide the metrological foundation for incorporating the Abdo signal into downstream deep learning applications. Building upon this foundation, our previous work demonstrated that these respiratory signals can be effectively integrated into an AI-based framework for automated sleep apnea screening, AHI estimation, and sleep apnea severity classification [18].

Option 2 (More cautious)

...These findings provide the metrological foundation for the use of the Abdo signal in downstream deep learning applications.

Option 3 (My favorite for your paper)

...These findings establish the technical foundation that supports the use of the Abdo signal in downstream deep learning applications.

Option 4 (Emphasizing enablement)

...These findings establish the technical foundation for subsequent AI-based analysis of the Abdo signal.

I actually prefer Option 3, because it is appropriately cautious:

These findings establish the technical foundation that supports the use of the Abdo signal in downstream deep learning applications.

It does not claim the signal is already "reliable," but instead says that your engineering evaluation provides evidence supporting its use in later AI models. That wording is well aligned with the moderate performance reported in your study and should be less likely to draw criticism from reviewers.

2. **Gemini 3.6 Flash**

**User Prompt:**

please rewrite this in inherent, concise, and easy to understand [A total of thirty-seven recordings were collected for analysis. Two recordings were excluded due to poor data quality from the Soomirang device: one with missing accelerometer data and another with sampling time errors. One recording was excluded due to severe hardware-related connection failures of the thoracic RIP belt. Technical inspection of these omitted sessions revealed prolonged flatlining and high-amplitude contact jitter (impulse noise artifacts) caused by sensor detachment or cable disconnections during unconstrained home monitoring. These reference-side failures were unrelated to patient clinical metrics (such as BMI or apnea severity).]

**AI Response:**

Here is a rewritten version that is highly coherent, concise, and easy for both readers and reviewers to follow. It clearly separates the exclusions by device so the source of the technical failure is immediately obvious.

Revised Manuscript Text

Of the 37 recordings collected, three were excluded due to technical failures during unconstrained home monitoring:

- Abdomen Sensor (Soomirang Device) Failures ($n=2$): One recording was omitted due to missing accelerometer data, and another due to critical sampling time errors.
- Reference System (Thoracic RIP Belt) Failure ($n=1$): One recording was excluded because of severe hardware connection loss. Technical inspection revealed prolonged signal flatlining and high-amplitude contact jitter (impulse noise artifacts) caused by sensor detachment or cable disconnection. This reference-side failure was purely technical and entirely unrelated to patient clinical metrics, such as BMI or apnea severity.

Why this works better:

- Immediate Clarity: Grouping the exclusions into bullet points by device makes it instantly clear *which* sensor failed and *why*.
- Better Flow: It flows logically from the total number ($37$) down to the specific technical reasons for the exclusions ($2+1=3$).
- Maintains Rigor: It preserves the technical defense explaining that the reference belt failure was an instrumentation issue rather than a patient-related limitation.

**User Prompt:** No, write in a paragraph

Here is the revised text rewritten into a smooth, cohesive paragraph that maintains full technical clarity and flows naturally:

Of the 37 recordings collected for analysis, three were excluded due to technical failures during unconstrained home monitoring. Specifically, two recordings were omitted due to data quality issues originating from the abdomen-worn Soomirang device, where one session suffered from missing accelerometer data and another exhibited critical sampling time errors. The third exclusion was caused by a severe hardware-related connection failure of the reference thoracic RIP belt; a technical inspection of this omitted session revealed prolonged signal flatlining and high-amplitude contact jitter (impulse noise artifacts) resulting from sensor detachment or cable disconnection. This reference-side failure was purely technical and entirely unrelated to patient clinical metrics, such as BMI or apnea severity.
